# Four hour ambulation after angioplasty is a safe practice method

**Published:** 2010

**Authors:** Mahin Moeini, Fatemeh Moradpour, Sima Babaei, Mohsen Rafieian, Alireza Khosravi

**Affiliations:** * Department of Medical Surgical Nursing, School of Nursing and Midwifery, Isfahan University of Medical Sciences, Isfahan, Iran; ** School of Nursing and Midwifery, Isfahan University of Medical Sciences, Isfahan, Iran; *** Department of Operating Room Nursing, School of Nursing and Midwifery, Isfahan University of Medical Sciences, Isfahan, Iran; **** Assistant Professor of Cardiology, School of Medicine, Isfahan University of Medical Sciences, Isfahan, Iran

**Keywords:** Angioplasty, getting out of bed, removing the sheet, femoral arterial sheet, rest in bed

## Abstract

**BACKGROUND:**

During the last 3 decades, there were increasing tendency towards angioplasty because of its benefits. But, this procedure has its acute problems like bleeding and formation of hematoma in the removal place of the sheet. Based on researchers’ clinical experiences, patients need a time of 8-12 hours for bed rest after coronary angioplasty. Recognizing desirable time for bed rest after angioplasty and remove the arterial sheet forms the foundation of related researches in the world. Getting out of bed soon after angioplasty, causes more comfortable feelings, less hospitalization period, fewer side effects of prolonged bed rest and less hospitalization expenses. Regarding less time for bed rest after angioplasty, the aim of this study was to assess the effect of the time of getting out of bed after angioplasty on the complications after removing the sheet in coronary angioplasty patients.

**METHODS:**

This was an experimental clinical study conducted in one step and two groups. Samples were included 124 angioplasty patients (62 in each group) who were chosen randomly from the CCU of Shahid Chamran hospital of the Isfahan University of Medical Sciences in 2007. Data were gathered by observing and evaluating the patients, using a questionnaire and a checklist. After angioplasty, patients from the intervention group were taken out of bed in 4 hours and patients from the control group were taken out of bed in 8 hours. After taking out of bed, patients were examined for bleeding and formation of hematoma in the place of taking out the arterial sheet. Data were analyzed using descriptive and inferential statistics via SPSS software.

**RESULTS:**

Results showed no meaningful difference between the two groups after getting out of bed (p > 0.05) regarding relative frequency of bleeding (p = 0.50), formation of hematoma (p = 0.34) and average diameter of hematoma (p = 0.39).

**CONCLUSIONS:**

Results of this study showed that reducing the bed rest time to 4 hours after removing the arterial sheet of size 7 do not increase bleeding and formation of hematoma in the removal place of the sheet. So, those angioplasty patients who do not have critical clinical condition and their vital symptoms are stabilized will be able to get out of bed 4 hours after removing the sheet.

Acute heart attack is a life threatening condition which could be distinguished by formation of a local necrotic area in myocardium. Every year, about one million and a hundred thousand Americans face heart attack. So, heart attack is the main reason of death in America and causes 529,000 deaths every year.^1^ In the beginning of 20^th^ century, cardiovascular diseases were responsible for less than 10% of deaths but by the beginning of 21^st^ century, cardiovascular diseases caused half of the deaths in the world and 25% of the in developing countries.^2^ In Iran, cardiovascular diseases have turned into an increasing problem. According to statistics, cardiovascular diseases caused 27% and 37% of deaths in 1979 and 1990, respectively. Then, every year cardiovascular diseases kill 150,000 Iranian population.^3^ Until 1997, coronary bypass surgery was the only method for treating coronary artery diseases. From that time, other methods like balloon holding cutter, stent, and atherectomy tools were used for repairing coronary artery. Nowadays, between 800 thousands to a million of angioplasties and 400 thousands of coronary bypass surgeries are being conducted every year.^4^

In the past 3 decades, there was an increasing tendency towards angioplasty because of its benefit but this procedure has its own acute problems like bleeding and formation of hematoma in the removal place of the arterial sheet.^5^ Frequency of side effects of the removal place of the arterial sheet in femoral artery after angioplasty based on diagnosis protocol varies between 1.5% to 18% and is related to the size of the sheet, the amount of prescribed anticoagulant and the length of remaining sheet in the artery.^6^ Coronary angioplasty is a nonsurgical method to increase the diameter of atherosclerotic coronary arteries by using balloon holding cutters. Cutters usually reach the tightened coronary artery through femoral artery after installing the arterial sheet.^7^

Leaving bed soon after angioplasty could increase comfortable feelings, lessen hospitalization time, lessen effects of long bed rest, and lessen the expenses. Supplying a safe and healthy protocol for the time of leaving bed after angioplasty improves patient’s comfort and safety. Reduction of bed rest time after procedure makes more patients to be ready for discharge from hospital. So, it can reduce hospital’s expenses and make room for treatment of patients with critical conditions.^8^ Boztosun et al took patients out of bed 2 hours after removing the artery angioplasty sheet from the skin. In that study, there were 8 cases (2.3%) who reported bleeding after taking out of bed. Considering the side effects related to the removal place of the sheet in conducted researches, their study showed that leaving the bed after 2 hours is a safe method.^9^ The results of Keeling et al study showed only 1 case of bleeding (2%) among patients of intervention group (left the bed 4 hours after removing the sheet) and there was no meaningful difference between them and the control group (who left the bed 6 hours after removing the sheet) regarding occurrence of bleeding.^10^ Based on researchers’ experiences in the CCU on taking care of angioplasty patients, they need 8-12 hours of bed rest after this procedure; this timing includes the time of angioplasty, the time that must be passed before removing the sheet from femoral artery, and the time that must be passed to make sure there are no bleeding and formation of hematoma.

Reviewing different hospitals’ policies, it could be understood that the desirable time for bed rest after removing the arterial sheet is not specified. Recognizing desirable time for patients to rest in bed after angioplasty and removing the arterial sheet in a way that they could move safely was the basic foundation of related researches; so, that services would be supplied with high quality and standards. Search for finding a care service based on observation is one of the most essential steps to create high quality services for patients.

Considering reduction of bed rest time after angioplasty, we decided to conduct an investigation to assess the effects of time of leaving the bed after removing the arterial sheet in coronary angioplasty patients.

Study’s special aims included the comparison of the following variables between intervention and control groups following removing the femoral sheet after angioplasty: relative frequency of occurrence and severity of bleeding, relative frequency of formation of hematoma and average diameter of hematoma.

## Methods

This was an experimental random clinical trial conducted in one step and two groups. Understudied samples were patients hospitalized in the CCU and cardiac ward of Shahid Chamran hospital of the Isfahan University of Medical Sciences in 2007 to have elective angioplasty and had inclusion criteria. First, sampling was done using simple sampling method. Then, they were randomly divided into two groups of intervention (odd admission numbers) and control (even admission numbers) and this was continued until the number of samples was enough. Considering other studies, the number of samples required for this study was considered 124 patients (62 per group). Sampling was done from June to October, 2007.

Inclusion criteria were as having elective angioplasty, conducting angioplasty through femoral artery, formation of homeostasis after removing the sheet by pressure of hand, using the arterial sheet of size 7, normal results of coagulation tests (PT, PTT, and INR) before angioplasty, the angioplasty done by the same cardiologist, and the patient agreement to participate in the study. Exclusion criteria included the history of coagulation and bleeding diseases, the history of surgery on femoral or iliac artery, history of heart attack or unstable angina during the study, extra-heparin administration during angioplasty, bleeding or formation of hematoma before removing the sheet or getting out of bed, long PTT before removing the sheet that delayed the removal, heparin administration after angioplasty, and unwillingness for getting out of bed or participate in the study.

Data were gathered by observing and evaluating the patients using a two part questionnaire and a checklist. The first part included 10 questions on demographic data and medical history and the second part included 39 questions which was the checklist for evaluating the patient before angioplasty, during angioplasty, at the time of going into the ward, before removing the femoral arterial sheet, and before and after getting out of bed. Required evaluation was done using the questionnaire before going for angioplasty. After the installation of size 7 arterial sheet, all of the samples received the same dose of heparin (72 to 100 units per each kilogram of body weight) during angioplasty to reach the active coagulation time of 205 to 300 seconds.^11^ Condition of the place of the sheet leaking of blood, bleeding, and formation of hematoma, and also dorsal tibial and metatarsus pulse were evaluated before leaving angioplasty ward, after acceptance in CCU, before removal of the femoral sheet, and after getting out of bed. Controlling the part that included the arterial sheet was done every 15 minutes for the first 2 hours after angioplasty, and then every 1-2 hours until the removal of the sheet. After removing the sheet, it was controlled again every 15 minutes for 3 hours.^5^ Then, after 24 hours it was checked to see if any delayed hematoma has been formed after getting out of bed.^6^ If there were bleeding and/or formation of hematoma before getting out of bed at any time, the samples were excluded from the study. For removing the sheet, INR, PTT and PT were controlled 3 hours after angioplasty. Four hours after angioplasty, a resident removed the sheet in a sterile situation. Hand pressure was applied for 15 minutes to make homeostasis.^12^ A pressure dressing was put on the removal place of the sheet. After the removal of the sheet, patients absolutely rested for 4 hours in intervention group and 8 hours in control group (after formation of homeostasis, they put sand bag on the patient for the period they were laid back in a straight position. In the last hour, they laid in bed while their head was in the position of 30-45 degrees). Both groups were controlled for bleeding, formation of hematoma, and dorsal tibial and metatarsus pulse before getting out of bed. If they did not have any vascular complications, they got out of bed and walked a distance of 3 meters. If there were no problems, they were allowed to walk for 3 more meters. Then, they were returned to bed for resting.^12^ If there was any bleeding after getting out of bed, the interval between getting out of bed and bleeding and the severity of bleeding based on the reduction of hemoglobin was measured. For measuring the reduction of hemoglobin, it was controlled 4 hours after the start of bleeding.^13^ The severity of bleeding was determined based on reduction of hemoglobin (mild bleeding: 1-3 grams reduction in one dl, medium bleeding: 3-5 grams reduction in one dl, severe bleeding: more than 5 grams reduction in one dl).^14^ Immediate aids after bleeding were bed rest and pressing the femoral artery with hand. Need of transfusion or other recommended interventions by physicians were recorded in the checklist. In the case of formation of hematoma, the interval between the hematoma formation and getting out of bed and the biggest hematoma diameter in cm were measured and recorded.

For validity of data gathering tools, content validity method was used. To make the study reliable, since the skill of cardiologist has an important role in complications after angioplasty, all of the angioplasty cases that were done by the cardiologist were included in the study. Required tests were done by two laboratory technicians. All of the tests were done in the laboratory of Shahid Chamran hospital. In this study, independent variable was the time of getting out of bed and dependent variables were occurrence and severity of bleeding and formation and size of hematoma. Gathered data were analyzed using and descriptive and inferential statistics via SPSS software.

The ethical committee of the Isfahan University of Medical Sciences approved the study.

## Results

Fisher exact test showed that background variables such as gender, age, body mass index (BMI), the history of high blood pressure, hyperlipidemia, cigarette smoking and diabetes were not different between the intervention and the control groups.

Considering the first and second aims of the study, only one mild bleeding was seen among cases. Results of accurate Fisher test with p = 0.50 showed no meaningful difference between both groups regarding relative frequency of occurrence of bleeding after getting out of bed. Formation of hematoma was seen in 3.22% in intervention group and in 6.44% in control group. Results of Fisher exact test with p = 0.34 showed no meaningful difference between both groups regarding relative frequency of formation of hematoma after getting out of bed. The average size of hematoma was 2 ± 0 in intervention group and 2.75 ± 1.5 in control group.

Results of t-student test with t = 1 and p = 0.39 showed no meaningful difference between both groups regarding the size of hematoma. Results of accurate Fisher test showed no meaningful difference between both groups considering the size of hematoma. There was no hematoma bigger than 5 cm in both groups.

## Discussion

Results of this study showed that taking patients out of bed in 4 hours after removing the sheet do not increase the occurrence of bleeding and formation of hematoma at the removal place. So, patients who had elective angioplasty and their vital symptoms are stabilized can leave the bed 4 hours after removing the sheet. The study of Boztosun et al showed 7 cases of bleeding during walking (2.1%) and one case of bleeding after walking (0.3%) that could be controlled using hand pressure. All of their patients left the bed after 2 more hours bed rest and showed no further complications. There were delayed bleeding in 3 patients (0.9%) after 48 hours that could be controlled by hand pressing and bed rest. New hematoma of about 1-2 cm was formed during the first week in 9 patients (2.6%). Findings of this study showed that complications of leaving the bed in 2 hours after coronary intervention with size 6 cutter through femoral artery are acceptable based on other studies’ findings.^9^

Pollard et al study conducted in two groups (leaving the bed after 2.5 hours and 4.5 hours) similar to the present study and showed no meaningful difference between the two groups considering bleeding and formation of hematoma. They said it is safe for patents to leave the bed after 2.5 hours bed rest.^15^

In the study of Vlasic et al, there were 99 patents in intervention group and 101 patients in control group. After random dividing, 9 patients (4.7%) showed formation of hematoma, 4 during the bed rest, and 5 after leaving the bed. But, there was no meaningful difference between the two groups regarding formation of hematoma. Also 3% of patients had bleeding that could be controlled by applying extrapressure. There was no meaningful difference between the two groups considering bleeding too.^16^

In Kleeng et al study, patients were randomly divided into two groups each consisting of 51 patients. Intervention group left the bed after 4 hours and control group left the bed after 6 hours. There was only one case of bleeding (2%) among intervention group. Based on the results of this study, time of leaving the bed after angioplasty was reduced to 4 hours in the Medical Center of the University of Virginia.^10^

Another study by Rosenstein et al was conducted to show the complications of the removal place. About 1.3% of the group who left the bed sooner than 2 hours and 16% of the group who left the bed later than 2 hours showed complications related to angioplasty. There was no case of big hematoma, retroperitoneal bleeding, and required blood transfusion. Results of this study showed no meaningful difference between the two groups regarding bleeding and formation of hematoma after leaving the bed.^17^

In the study of Koch et al, the frequency of occurrence of bleeding was 1.7%. There was bleeding in 4 patients while leaving the bed and in 1 patient 15 minutes after leaving. Report of 48-hour follow-up showed big hematoma in 3% of patients which means 9 patients had hematoma bigger than 5 cm. No need for blood transfusion or vascular reconstructive surgery was reported. Bleedings could be controlled by bed rest and applying extrapressure. No case had delayed bleeding or vascular complications. Also, leaving the bed in 4 hours after angioplasty was safe for patients and did not increase the risk of bleeding and formation of hematoma in comparison with leaving the bed after 8 hours.^5^

Patients endure a long-time bed rest during and after angioplasty. This condition is not tolerable for patients. Given the results of previous studies and the present study, researchers hope that physicians and nurses consider the recommended time for those patients with elective angioplasty and stabilized vital symptoms. Also, the researchers hope that results of this study be used for giving more information to health care personnel who work with coronary angioplasty patients for teaching students and retrained nurses, so it could improve patients’ comfort and safety.

The authors declare no conflict of interest in this study.
